# Tanshinone IIA Inhibits Osteosarcoma Growth through a Src Kinase-Dependent Mechanism

**DOI:** 10.1155/2021/5563691

**Published:** 2021-06-30

**Authors:** Chao Hu, Xiaobin Zhu, Taogen Zhang, Zhouming Deng, Yuanlong Xie, Feifei Yan, Lin Cai

**Affiliations:** ^1^Department of Spine Surgery and Musculoskeletal Tumor, Zhongnan Hospital of Wuhan University, No. 169th, Donghu Road, Wuchang District, Wuhan 430071, Hubei, China; ^2^Department of Orthopedics, The Second Affiliated Hospital of Hubei Polytechnic College, Dongfeng Road, Daye 435100, Hubei, China

## Abstract

**Introduction:**

Osteosarcoma is a malignant tumor associated with high mortality rates due to the toxic side effects of current therapeutic methods. Tanshinone IIA can inhibit cell proliferation and promote apoptosis *in vitro*, but the exact mechanism is still unknown. The aims of this study are to explore the antiosteosarcoma effect of tanshinone IIA via Src kinase and demonstrate the mechanism of this effect.

**Materials and Methods:**

Osteosarcoma MG-63 and U2-OS cell lines were stable transfections with Src-shRNA. Then, the antiosteosarcoma effect of tanshinone IIA was tested *in vitro.* The protein expression levels of Src, p-Src, p-ERK1/2, and p-AKt were detected by Western blot and RT-PCR. CCK-8 assay and BrdU immunofluorescence assay were used to detect cell proliferation. Transwell assay, cell scratch assay, and flow cytometry were used to detect cell invasion, migration, and cell cycle. Tumor-bearing nude mice with osteosarcoma were constructed. The effect of tanshinone IIA was detected by tumor HE staining, tumor inhibition rate, incidence of lung metastasis, and X-ray.

**Results:**

The oncogene role of Src kinase in osteosarcoma is reflected in promoting cell proliferation, invasion, and migration and in inhibiting apoptosis. However, Src has different effects on cell proliferation, apoptosis, and cell cycle regulation among cell lines. At a cellular level, the antiosteosarcoma effect of tanshinone IIA is mediated by Src downstream of the MAPK/ERK and PI3K/AKt signaling pathways. At the animal level, tanshinone IIA played a role in resisting osteosarcoma formation by Src downstream of the MAPK/ERK and PI3K/AKt signaling pathways.

**Conclusion:**

Tanshinone IIA plays an antiosteosarcoma role *in vitro* and *in vivo* and inhibits the progression of osteosarcoma mediated by Src downstream of the MAPK/ERK and PI3K/AKt signaling pathways.

## 1. Introduction

Osteosarcoma is the most common malignancy of the skeletal system in adolescents. Currently, the standard neoadjuvant therapy is combined with surgery and chemoradiotherapy, but the 5-year survival rate does not exceed 58% [1]. The occurrence and progression of osteosarcoma result from the accumulation of polygenic abnormalities involving multiple signaling pathways [2–4]. Osteosarcoma lacks specific markers, and therefore, finding a common target in various signaling pathways is a challenge in current research.

Recent studies have found that Src protein is associated with multiple signaling pathways and is the central hub of multiple signaling pathways, such as MAPK and PI3K pathways. Abnormal expression or activation of Src can promote the occurrence and development of malignant tumors [5, 6]. In our previous study [7], we found that the expression levels of Src were negatively correlated with the prognosis of patients with osteosarcoma, suggesting that Src may play a key role in osteosarcoma.

The drugs targeting the Src family are investigated, such as dasatinib. It has been demonstrated that dasatinib could inhibit tumor biological functions in prostate cancer [8], lung cancer [9], and breast cancer [10]. In addition to the continued development of targeted inhibitors, the search for natural and efficient targeted drugs with nontoxic side effects has become a hotspot. Natural traditional Chinese medicine is widely known domestically and abroad for its wide range of curative effects, accuracy, and low toxicity. Tanshinone IIA is a fat-soluble active ingredient extracted from the traditional Chinese medicine *Salvia miltiorrhiza*, which has the function of protecting cardiovascular and nerve cells. Our previous *in vitro* studies [11] found that tanshinone IIA inhibited cell proliferation and promoted apoptosis by regulating the expression level of Bcl-2/Bax in osteosarcoma cells. The inhibition of cell migration and invasion was concentration- and time-dependent. Tanshinone IIA-regulated Bcl-2/Bax protein exists in Src-mediated MAPK and PI3K signaling pathways [12, 13]. Therefore, this study will elaborate on the Src-mediated antiosteosarcoma mechanism of tanshinone IIA.

## 2. Materials and Methods

### 2.1. Chemicals and Antibodies

Lentivirus (Biovector, China), Src-shRNA (GenePhama, China), Cy3 Conjugated Rabbit Anti-Goat IgG Secondary Antibody (Boster, China), 98% tanshinone IIA (Sigma-Aldrich, USA), Src antibody (#2109) (Cell Signaling, Inc., USA), p-Src antibody (#2101) (Cell Signaling, Inc., USA), p-ERK1/2 antibody (#4695) (Cell Signaling, Inc., USA), and p-AKt antibody (#4059) (Cell Signaling, Inc., USA) were used. BLAB/C-nu/nu nude mice purchased from China Vital River, Inc. (Beijing, China), were used in this study.

### 2.2. Cell Culture

The human osteosarcoma cell lines U2-OS and MG-63 were obtained from the Type Culture Collection of Chinese Academy of Sciences (China). The cells were cultured in DMEM (Hyclone, USA) containing 100 *μ*g/mL penicillin, 100 *μ*g/mL streptomycin, and 10% fetal bovine serum (Invitrogen, USA) in a humidified incubator with 5% CO_2_ at 37°C.

### 2.3. Cell Proliferation Assay

Osteosarcoma cells were seeded in 96-well microplates. After exposure to different treatments for 24 h, cell proliferation was then measured using a Cell Counting Kit-8 (Dojindo Molecular Technologies, Japan). Cell proliferation was performed at 450 nm using a microplate reader (TECAN SPECTRA, Switzerland), and the relative inhibition rate was analyzed according to our previous study [11].

### 2.4. Apoptosis Assay

In brief, 1 × 10^6^ cells were harvested following trypsinization and centrifugation, then washed with PBS, and resuspended in binding buffer. The cells were incubated with 2 *μ*L Annexin V-FITC and 2 *μ*L propidium iodide (PI) (Sungene Biotech, China) for 15 min in the dark. Apoptotic cells were performed by flow cytometry (Beckman Coulter, USA).

### 2.5. Cell Cycle Analysis

In brief, 1 × 10^5^ cells per well were cultured in 6-well plates and then treated with 0 or 30 *μ*mol/L tanshinone IIA for 24 h. Following the treatment, we collected the cells and assessed them using the Cell Cycle Detection Kit (Boster, China). Moreover, 5 *μ*L PI was added after incubation with RNase for 20 min at 37°C, followed by a 20 min incubation at room temperature in the dark. Then, the samples were analyzed by flow cytometry and ModFit LT 3.0 cell cycle analysis software (Becton Dickinson, USA).

### 2.6. Cell Scratch Assay

Osteosarcoma cells were prepared in cell suspension in a 6-well plate with a concentration of 5 × 10^4^/mL and 2 mL of the cell suspension was added to each well for the routine culture. After the cells were confluent, we discarded the medium and used a 200 *μ*L tip to make scratches parallel to the edge of the culture plate on the bottom surface of each well. After the plates were delimited, they were rinsed with sterilized PBS three times. Observations and photographs were taken under an inverted microscope and four fields of view were randomly selected for each well. A serum-free medium was then used to continue the culture and the cells were observed and photographed again 24 hours later [14].

### 2.7. Cell Invasion Assay

We use modified chambers containing Transwell polycarbonate membranes (BD, USA) to perform the cell invasion assay. First, 5 × 10^5^ cells were seeded into the upper chamber (coated with 100 *μ*g/mL Matrigel). After incubation for 24 h at 37°C, the cells invading the lower surface were stained with 0.1% crystal violet. Five fields of view for each chamber were randomly selected for counting under a microscope.

### 2.8. Immunofluorescence Staining

Cells were cultured on coverslips overnight. After being treated for 24 h, cells were washed with PBS and then fixed in 4% paraformaldehyde for 15 min. Then, cells were washed twice with PBS and soaked in PBS with 0.5% Triton X-100 for 20 min. The coverslips were incubated for 30 min at room temperature with a primary antibody. After being washed with PBS three times, cells were incubated with Cy3 Conjugated Rabbit Anti-Goat IgG Secondary Antibody. The nuclei were stained with DAPI for 5 min, and the images were photographed under a fluorescence microscope (DX51, Olympus, Japan).

### 2.9. Western Blotting

Cells were harvested and lysed in cell lysis buffer (Boster, China). The lysates were centrifuged at 15,000 × g for 10 min at 4°C. The sample protein was separated on SDS-PAGE and transferred onto a nitrocellulose membrane. The membrane was incubated with primary antibodies overnight. Then, the membrane was incubated with secondary antibodies conjugated with HRP at room temperature for 2 h. The proteins were visualized with an enhanced chemiluminescence (ECL) system (Boster, China). The band intensity was analyzed using Quantity One software (Bio-Rad, USA).

### 2.10. Nude Mouse Tumor Formation

Twenty female BLAB/C-NU/nu nude mice, aged 4∼6 weeks and weighing 21.16 ± 2.17 g, were used in the experiment. The mice were fed in sterile air filter enclosures, with a constant temperature (26∼28°C) and constant humidity (40%∼60% relative humidity). The whole experiment achieved specific free (SPF) conditions. We prepared the cell suspension with 1 × 10^7^ cells/piece with a 26G syringe. We randomly selected nude mice and, after disinfecting its left hind leg and keeping the knee flexion at 90°, inserted the syringe needle into the tibia bone marrow cavity perpendicular to the tibia platform joint surface, and then injected the cell suspension. Routine feeding was observed under the SPF environment after injection. Generally, tumorigenesis is identified at 2–3 weeks when the local tumor mass size was about l cm^3^. Tumor growth status was determined as follows [15]: poor tumor growth was defined as an average tumor weight of  < *l* g or if the tumor weight was <400 mg in mice with 20% overall tumor weight. Nude mice with poor tumor growth that met these criteria were considered a failed model and were excluded. Mice were sacrificed by cervical dislocation. The animal experiment was approved by the Institutional Animal Care and Ethical Committee of our hospital. All of the procedures were performed in accordance with standards of laboratory animal care.

### 2.11. Treatment of Nude Mice

We successfully modeled a total of 18 nude mice with osteosarcoma *in situ*. The test was conducted after calculating tumor weight and size, then randomly dividing the mice into two groups, followed by three days of SPF feeding. The experimental group was given tanshinone IIA gavage (10 mg/kg/d) for two weeks [16], while the control group was given normal saline every 5 mg for two weeks. We observed the following indices: (1) Mental state, diet, weight activity sensitivity, and other general conditions of the mice were evaluated. (2) Two perpendicular diameter measurements of the tumor, D1 and D2, were carried out every week; tumor volume and growth curves were calculated according to the following formula: *V* = 4/3 PI [1/4(*D*1 + *D*2)]2. (3) Osteosarcoma tissue was sampled, tumor mass was measured, and HE staining was performed four weeks after treatment. If any mice died during the treatment, we excised the tumor and performed pathological examination within 6 hours after death. (4) Tumors were weighed and tumor inhibition rate was calculated according to the following formula: (the average tumor weight of the control group-the average tumor weight of the experimental group)/the average tumor weight of the control group × 100. (5) A survival analysis chart for the two groups was created. (6) Western blots were performed to detect the expression of Src, p-Src, p-MAPK, p-ERK, p-PI3K, and p-AKt proteins in the tumor. (7) An X-ray was performed before and after treatment to assess the size of the tumor.

### 2.12. Statistical Analysis

All data are expressed as mean ± SEM. Statistical Product and Service Solutions (SPSS) version 18.0 was used for statistical analysis. Results were statistically significant if *P* < 0.05 using a two-tailed paired Student's *t*-test. We used log-rank tests and Kaplan–Meier survival curve analysis for survival analysis. All experiments were performed in triplicate.

## 3. Results

### 3.1. Effect of Tanshinone IIA on the Biological Behavior of Osteosarcoma Cells

To verify the effect of Src on osteosarcoma cells, Src-shRNA with GFP-labeled was stably transfected into osteosarcoma cells by lentiviral transfection. After the successful construction of stable Src-shRNA transfected cells, we found that the biological behavior of osteosarcoma cells was inhibited by Src-shRNA transfection. All the results are shown in Supplementary Figures S1–S9.

**Figure 1:**
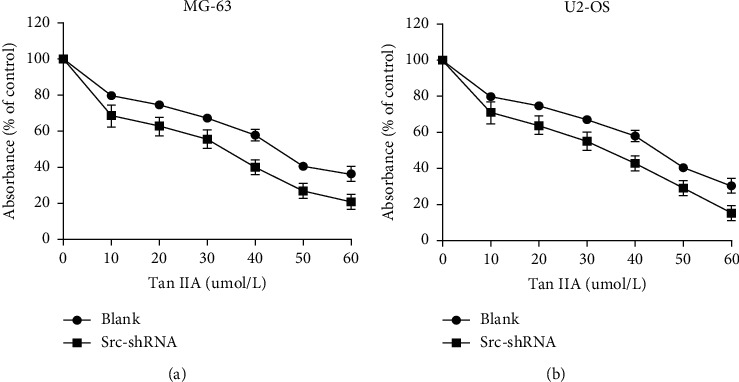
Tanshinone IIA inhibits MG-63 cell line proliferation measured by the CCK-8 assay. (a) MG-63 cell line; (b) U2-OS cell line.

**Figure 2:**
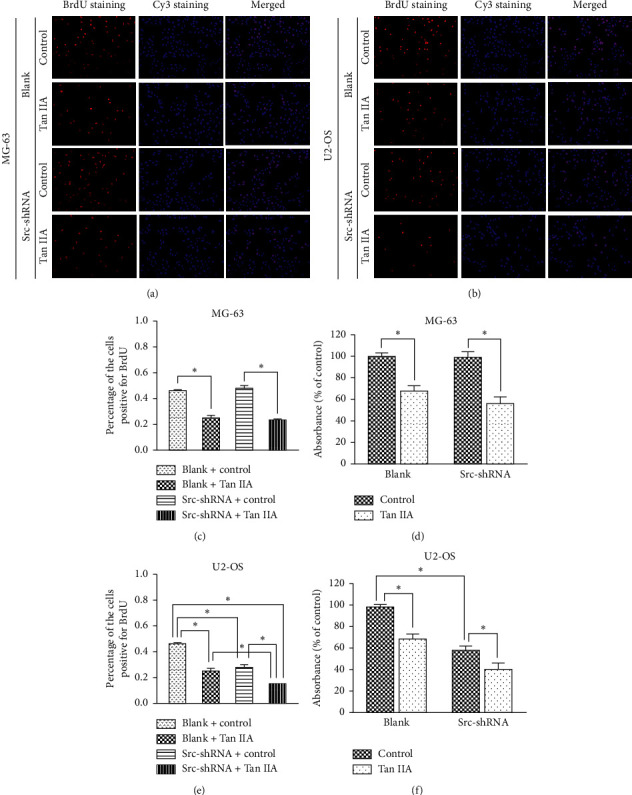
The effect of tanshinone IIA on osteosarcoma cell proliferation: (a, c) proliferation of the MG-63 cell line was inhibited by tanshinone IIA measured by immunofluorescence against BrdU; (b, e) proliferation of the U2-OS cell line was inhibited treated by Src-shRNA or tanshinone IIA, and they had a synergetic function to inhibit cell proliferation measured by immunofluorescence against BrdU; (d, f) tanshinone IIA induced inhibition of osteosarcoma cell proliferation in MG-63 and U2-OS cell line measured by the CCK-8 assay (^*∗*^*P* < 0.05; ×200).

**Figure 3:**
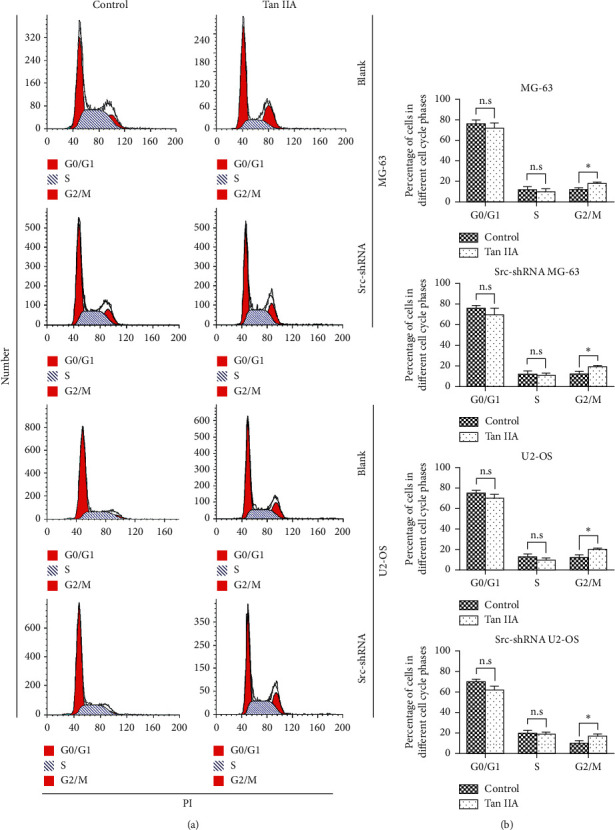
The effect of tanshinone IIA on the osteosarcoma cell cycle measured by flow cytometry. (a) Cell cycle analysis of MG-63 and U2-OS cells; (b) quantification of each cell cycle phase in MG-63-, U2-OS-, and Src-shRNA-transfected cells (^*∗*^*P* < 0.05).

**Figure 4:**
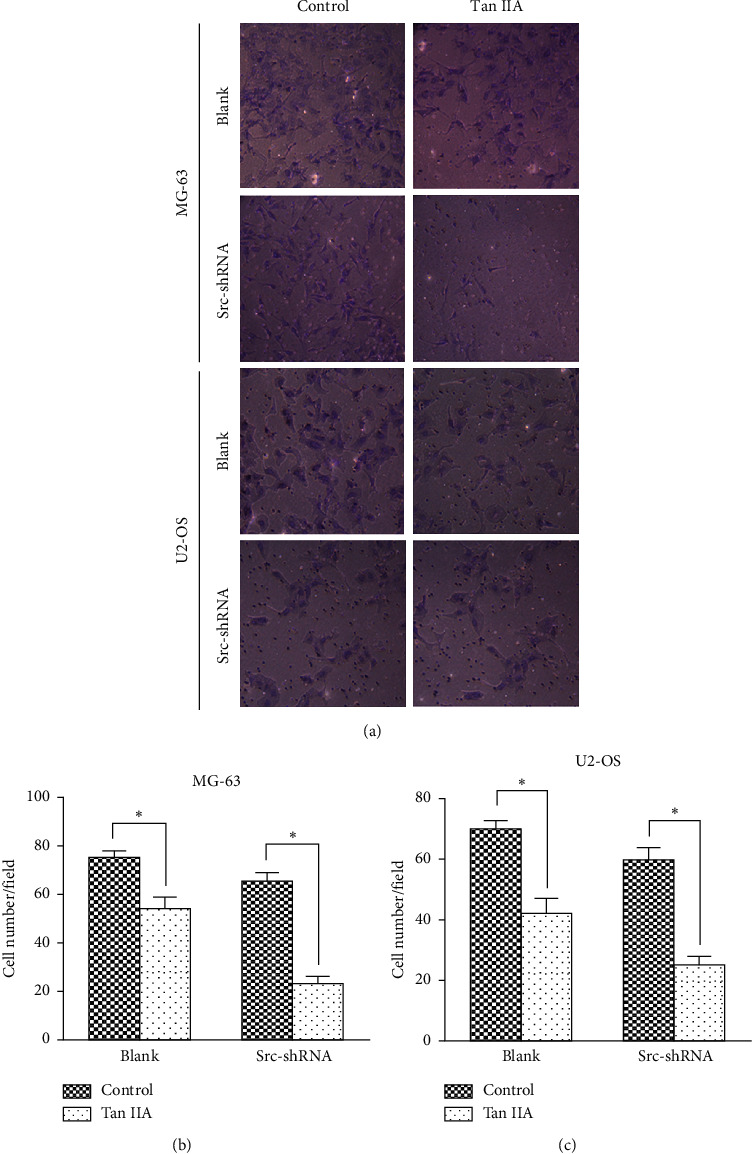
Tanshinone IIA inhibited invasion in MG-63 and U4-OS cells measured by Transwell. (a) The view of MG-63 and US-OS cells in each group under a microscope (×400). (b, c) Quantification of each cell number in MG-63 and US-OS cells in each group shown in (a). Five fields of view for each chamber were randomly selected for counting under a microscope.

**Figure 5:**
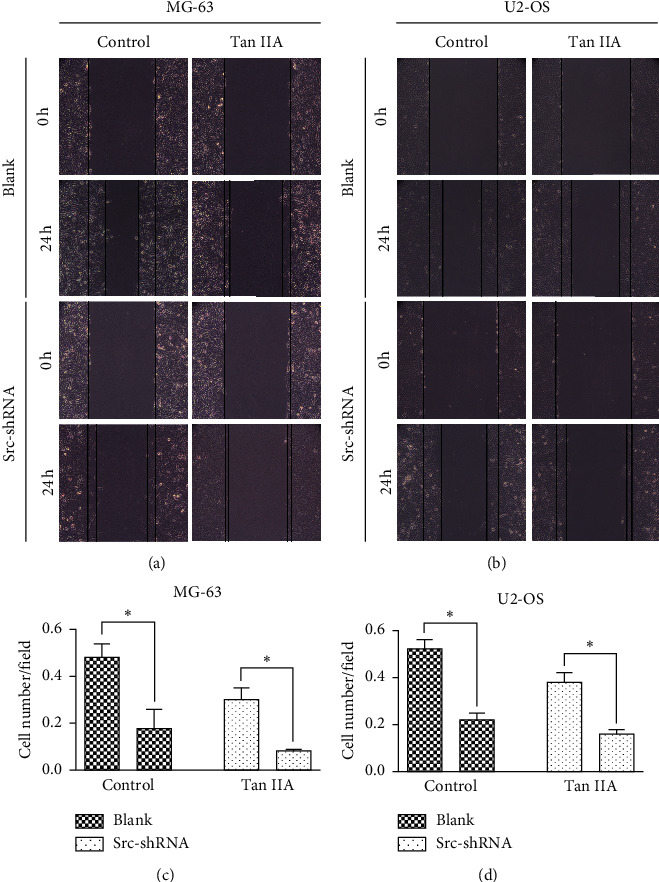
Tanshinone IIA inhibited migration of osteosarcoma cells. (a, b) The pictures of MG-63 and U5-OS cell lines under the electron microscope measured by the cell scratch assay (×500); (c, d) quantification of relative migration ability of MG-63 and US-OS cells in each group shown in (a) and (b) (^*∗*^*P* < 0.05).

**Figure 6:**
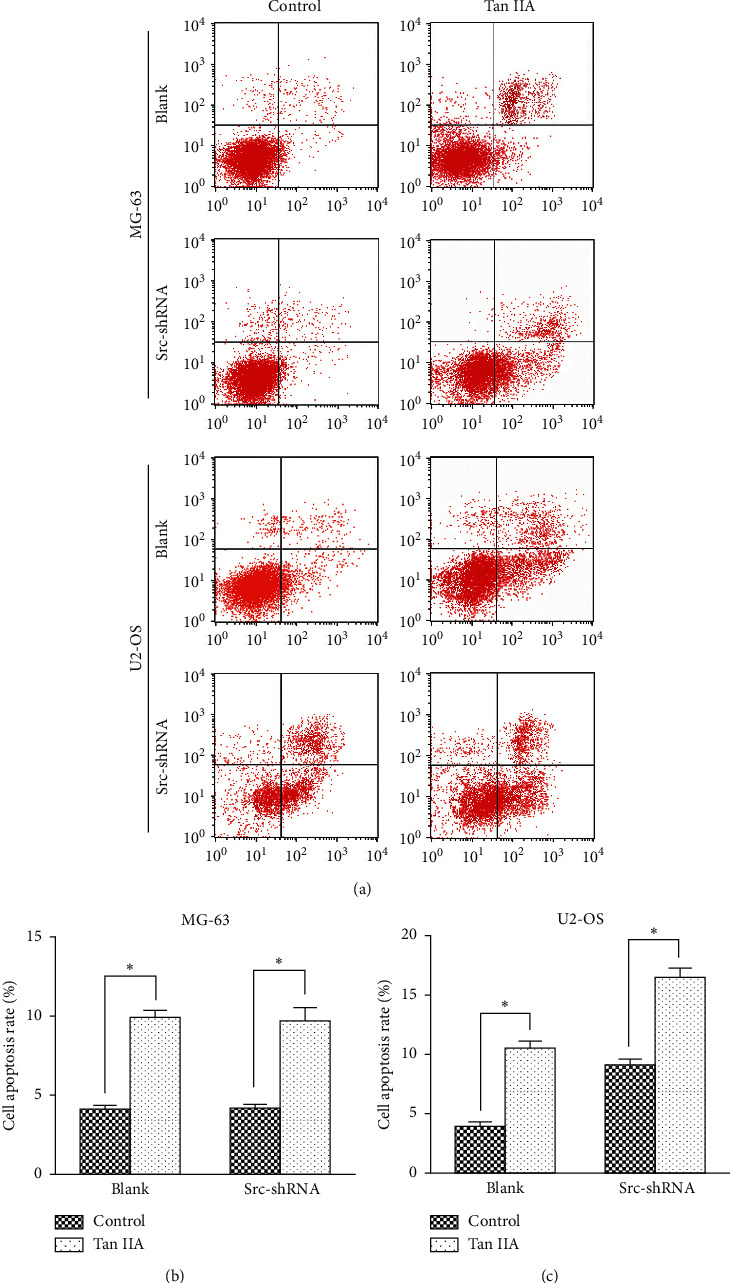
The effect of tanshinone IIA on apoptosis of osteosarcoma cells measured by flow cytometry. (a) The representative images of Annexin V and propidium iodide (PI) staining of MG-63 and U6-OS cells. (b) Quantification of cell apoptosis rate shown in (a). Data are presented as mean ± SD of 3 independent experiments (^*∗*^*P* < 0.05).

We determined the effect of tanshinone IIA on osteosarcoma cell proliferation using the CCK-8 assay, after which we determined the optimal concentration 24 hours after treatment of osteosarcoma cells. The results show that the IC50 of tanshinone IIA-treated cells were 39.2 *μ*mol/L in untransfected MG-63 cells, 25.2 *μ*mol/L in stable Src-shRNA-transfected MG-63 cells after 24 h treatment, 39.3 *μ*mol/L in untransfected U2-OS cells, and 26.7 *μ*mol/L in stable Src-shRNA-transfected U2-OS cells (Figure 1). The above concentrations were generally between 25 and 40 *μ*mol/L; therefore, we chose 30 *μ*mol/L tanshinone IIA as the standard concentration for subsequent experiments. We found that the tanshinone IIA could induce inhibition of osteosarcoma cell proliferation (Figure 2; *P* < 0.05). Compared with untransfected osteosarcoma cells, tanshinone IIA was more effective in inhibiting cell proliferation in Src-shRNA-transfected U2-OS cells (Figures 2(b), 2(e) and 2(f); *P* < 0.05). The results obtained by the BrdU immunofluorescence luminescence method were the same as those obtained by the CCK-8 assay. The results show that tanshinone IIA induced G2/M phase cell cycle arrest of MG-63 and U2-OS cells (Figure 3). Transwell experiments showed that tanshinone IIA significantly reduced the invasion ability of both MG-63 and U2-OS cell lines (Figure 4). Cell scratch assay showed that tanshinone IIA inhibited cell migration more significantly in the Src-shRNA transfection group than in the untransfected group (Figure 5). The results show that tanshinone IIA promotes apoptosis of osteosarcoma MG-63 and U2-OS cell lines (Figure 6).

### 3.2. Tanshinone IIA Inhibited the Expression of Src/MAPK/ERK and Src/PI3K/AKt in Osteosarcoma Cells

**Figure 7:**
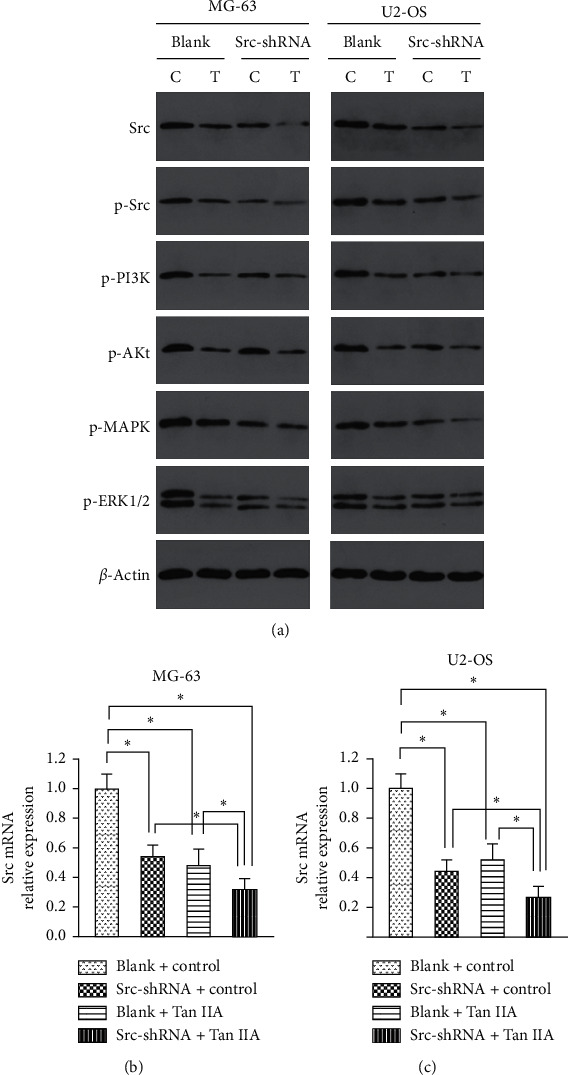
Tanshinone IIA inhibited expression of Src/MAPK/ERK and Src/PI3K/AKt. (a) The effect of tanshinone IIA on the expression of Src, p-Src, p-MAPK, p-ERK1/2, p-PI3K, and p-AKt in osteosarcoma cells measured by Western blot. (b, c) The effect of tanshinone IIA on the expression of Src mRNA in osteosarcoma cells measured by RT-PCR. C: control group; T: tanshinone IIA (^*∗*^*P* < 0.05).

After osteosarcoma cells were treated with tanshinone IIA, Western blot and RT-PCR analyses were performed. We found that tanshinone IIA can inhibit the expression of Src, p-Src, p-MAPK, p-ERK1/2, p-PI3K, and p-AKt proteins (Figure 7(a)) and Src mRNA in the above two cell lines (Figure 7(b)) (*P* < 0.05). Tanshinone IIA and Src-shRNA had a synergetic function to inhibit the proteins above.

### 3.3. Growth of Tumor-Bearing Nude Mice

**Figure 8:**
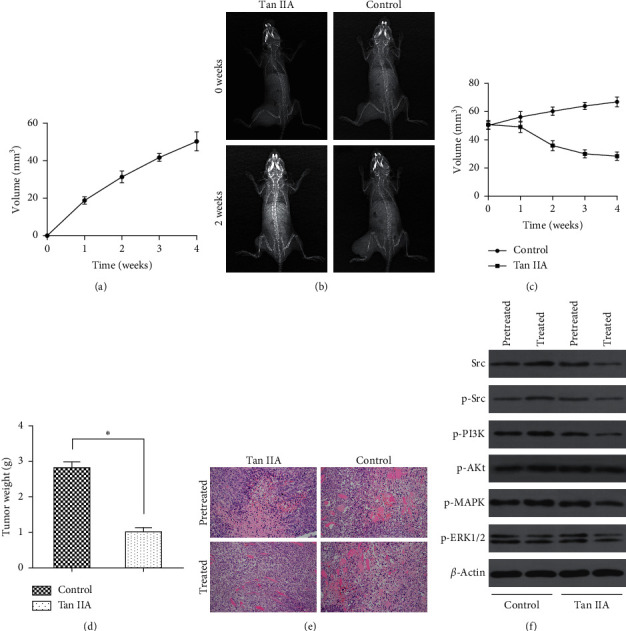
Tanshinone IIA can inhibit the growth of osteosarcoma in tumor-bearing nude mice. (a) Tumor growth curve of tumor-bearing nude mice after inoculation. (b) X-rays taken before and after tanshinone IIA treatment showed that the soft tissue shadow of the left hind limb of the experimental group was reduced compared with that of the control group. (c) Tumor growth curve showed that tanshinone IIA inhibited the tumor growth. (d) Weight measurements of the tumor following tanshinone IIA treatment. (e) Tanshinone IIA increased tumor cell differentiation and light cell nucleus measured by HE staining (×200). (f) Tanshinone IIA inhibited expression of Src, p-Src, p-MAPK, p-ERK1/2, p-PI3K, and p-AKt in osteosarcoma tissue measured by Western blot (^*∗*^*P* < 0.05; pretreated, the mice were not treated with tanshinone IIA or saline; treated, the mice were treated with tanshinone IIA or saline).

There was no statistical difference in body weight and other factors between the experimental and control groups for the total 18 nude mice for the experiment. After inoculation (Supplementary Figure S10), the mice showed gradually poorer mental state, decreased appetite, and emaciation, while the tumor continuously grew (Figure 8(a)).

Our observations showed the tumor section was gray-white and fishy, and the tumor tissue infiltrated and grew in the tibia and broke through the bone cortex to grow into the surrounding soft tissue. Sections of the fibula were also eroded and missing. After four weeks of tumor growth, the HE section showed partial tumor focal necrosis under the light microscope. X-ray examination revealed enlarged soft tissue shadow in the left hind limb, osteolytic destruction of the left tibia, and absence of the left fibula. The cells were of different sizes and shapes, showing round, polygonal, or spindle shapes, in addition to other forms. The nuclei were large and the nucleoli were clear (Supplementary Figure S11).

### 3.4. Tanshinone IIA Can Inhibit Growth of Osteosarcoma in Tumor-Bearing Nude Mice

X-rays taken before and after tanshinone IIA treatment showed that the soft tissue shadow of the left hind limb of the experimental group was reduced compared with that of the control group. A tumor growth curve was drawn after tanshinone IIA treatment (the first tanshinone IIA treatment was recorded at 0 weeks) (Figure 8(b)), indicating that the tumor in the experimental group shrank, while the tumor in the control group continued to slowly grow (Figure 8(c)). Four weeks after tanshinone IIA treatment, the tumor inhibition rate of tanshinone IIA was calculated based on tumor weight as 64.09% (Figure 8(d)). HE staining of the tumor showed that necrosis of some tumor foci was observed in the control group, with different cell sizes and morphologies, showing round, polygonal, or short fusiform and other forms. The cell nucleus was large and the nucleoli were clear. Pathological mitosis with different morphologies and obvious hyperchromatism of the nucleus were observed. In the experimental group, nuclear staining intensity decreased and some cell nuclei were reduced (Figure 8(e)).

### 3.5. Tanshinone IIA Inhibited Activation of Signaling Pathways in Tumor Tissue

Western blot was used to detect Src, p-Src, p-MAPK, p-ERK1/2, p-PI3K, and p-AKt proteins in osteosarcoma tissues of the two groups before and after treatment. The results showed that these proteins were inhibited in the experimental group compared with the control group (Figure 8(f)). The results suggest that tanshinone IIA inhibited Src-mediated activation of MAPK/ERK and PI3K/AKt signaling pathways in nude mice.

### 3.6. Tanshinone IIA Can Improve Survival of Tumor-Bearing Nude Mice

**Figure 9:**
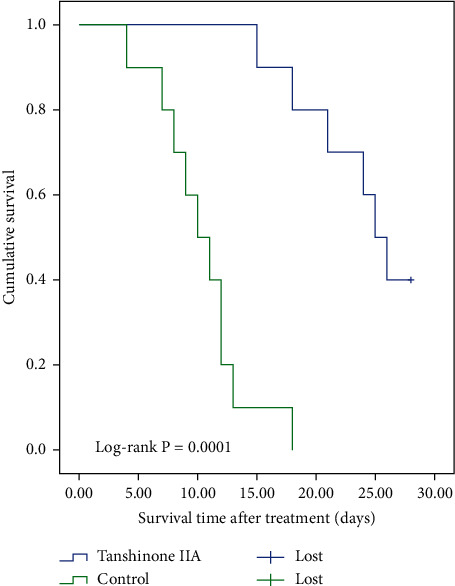
Postoperative survival curves in nude mice. Eighteen nude mice with osteosarcoma were divided into an experimental group (given tanshinone IIA) and control group (given normal saline). Survival analysis showed that the survival time of the experimental group was longer than that of the control group (*P* < 0.05).

We used Kaplan–Meier curves to analyze the total survival number with one variable. Different processing methods and survival time were statistically significant at *P* < 0.05 (log-rank test, Figure 9). Survival analysis showed that the survival time of the tanshinone IIA group was longer than that of the control group (*P* < 0.05).

## 4. Discussion

Osteosarcoma is a highly malignant musculoskeletal cancer that tends to occur in adolescents, the tumor recurrence rate is still higher after limb salvage or amputation, and chemoradiotherapy is not effective for some patients and with many side effects [17]. Therefore, finding new targeted drugs or adjuvant therapies has always been a hot topic.

Tanshinone IIA has been widely used in clinical practice in China, mainly to improve cardiovascular circulation. In recent years, tanshinone IIA has been found to play a role in antitumor function [18–24]. It can induce apoptosis of prostate cancer and leukemia cells through PI3K/AKt signal pathway [25, 26] and induce the expression of miR-1 in mice after myocardial infarction through P38/MAPK pathway [27]. In addition, tanshinone IIA can inhibit osteoclast differentiation through Src [28].

The phosphorylation locus of c-Src includes the phosphorylation activation site at Tyr416 in the N-terminal tail and the negative phosphorylation site at Tyr527 in the C-terminal tail. C-Src kinase can be activated into the open conformation through the phosphorylation at Tyr416 or dephosphorylation at Tyr527 to act as an oncogene. As the Supplementary Materials show, inhibition of Src phosphorylation in the U2-OS cell line can induce apoptosis and cell cycle arrest and inhibit cell proliferation, but no significant effects on apoptosis, proliferation, and cell cycle were noted in the MG-63 cell line. Besides, tanshinone IIA and src-shRNA had synergistic effects on the proliferation of U2-OS but not on MG-63 cells. The reason may be that previous work has shown that the p53 gene in MG-63 cells (p53−/−) is inactivated, while the p53 gene in U2-OS is positive (p53+/+) [29]. Therefore, the Src-induced regulation of proliferation, apoptosis, and cell cycle may be related to p53 gene expression, as demonstrated by Fu and Shor [30, 31]. Jung et al. had demonstrated that the ability of Src inhibiting cell proliferation and inducing apoptosis depending on P53 [32]. Therefore, we speculated that tanshinone IIA may also play an antiproliferation role in osteosarcoma through nondependent Src or P53 signaling pathways. This inference has also been demonstrated by Ma that tanshinone IIA can inhibit the proliferation of MG-63 and promote apoptosis by inducing autophagy [33].

Src is the central hub of multiple signaling pathways and plays an important role in human physiological and pathological activities. The confirmed signal pathways are mainly as follows: Src/MAPK, Src/PI3K/AKt, Src/JAK2/STAT3, Src/EGFR, Src/FAK, and Src/PLC pathways [34–38]. PI3K/AKt and MAPK signal pathways are the two main downstream pathways of Src. The mitogenic and antiapoptosis effects led by the two signal pathways play an important role in improving human malignant tumor cells [39, 40]. In recent years, the study found that PI3K/AKt signal pathway exists in a variety of human tumors, including osteosarcoma expression disorders [41, 42]; the phosphorylated AKt (p-AKt) could influence its downstream target protein Bad (members of the family of the Bcl-2), caspase 9, and NF-*κ*B, thus regulating cell proliferation, cell differentiation, and apoptosis. In addition, activation of the PI3K/AKt signal pathway is also closely related to tumor angiogenesis, invasion, and metastasis, thereby affecting the prognosis of patients [43, 44].

The MAPK signaling pathway activates a series of signaling transduction molecules through a cascade reaction, which ultimately phosphorylates its downstream gene ERK. After phosphorylated ERK (p-ERK) entered the cell nucleus, the effector genes in the nucleus were activated. Then, the transcription of the target genes related to cell proliferation were finally activated, thus exerting the effect of promoting the proliferation, invasion, and migration of tumor cells. At present, activation of the MAPK pathway has been found in various tumor tissues, including osteosarcoma, gastric cancer, pancreatic cancer, ovarian cancer, and lung cancer [45–49]. Moreover, multiple proteins in MAPK/ERK pathways are involved in the effects of osteosarcoma [50–56].

Tanshinone IIA can induce cell cycle arrest, but the effects of tanshinone IIA on MG-63 and U2-OS cells are different in this study. The reason may be that the original proportions of each cell cycles of the two cell lines are different (mainly of S and G2/M phase), as described by Li et al. [57]; therefore, after being treated by tanshinone IIA, the cell cycle patterns of the two cell lines were also different, although both were arrested in the G2/M phase. In addition, the effects of tanshinone IIA on cyclins of MG-63 and U2-OS cells are also unclear, which need to be explored in future studies.

## 5. Conclusion

The oncogene role of Src kinase in osteosarcoma is reflected in promoting cell proliferation, invasion, and migration and inhibiting apoptosis. However, Src has different effects on cell proliferation, apoptosis, and cell cycle regulation among cell lines. At a cellular level, the antiosteosarcoma effect of tanshinone IIA is mediated by Src downstream of the MAPK/ERK and PI3K/AKt signaling pathways. At the animal level, tanshinone IIA played a role in resisting osteosarcoma formation by Src downstream of the MAPK/ERK and PI3K/AKt signaling pathways. Thus, tanshinone IIA plays an antiosteosarcoma role *in vivo* and inhibits the progression of osteosarcoma.

## Data Availability

All data, models, or codes generated or used during the study are available from the corresponding author upon request.

## Abbreviations

AKt: Protein kinase B
BrdU: Bromodeoxyuridine
CCK-8: Cell Counting Kit-8
ERK: Extracellular signal-related kinase
MAPK: Mitogen-activated protein kinase
MEK: Mitogen-activated protein kinase kinase-MAPKK
PI3K: Phosphatidylinositide 3-kinase.

## Supplementary Materials

Supplementary Materials. Supplementary Figure S1: GFP-labeling of stable Src-shRNA-transfected osteosarcoma cells. The cells were shown in green, and the nuclei labeled by DAPI were shown in blue. (a) MG-63 cell line; (b) U2-OS cell line (×200). Supplementary Figure S2: transfection with Src-shRNA into osteosarcoma cells affected the expression of Src mRNA measured by RT-PCR. The expression of Src mRNA decreased in the MG-63 and U2-OS cell line (NC, negative control. ^*∗*^*P* < 0.05). Supplementary Figure S3: the effect of transfection with on the expression of Src, p-Src, p-ERK1/2, and p-AKt in osteosarcoma cells measured by Western blot. The expressions of the proteins were inhibited (NC, negative control). Supplementary Figure S4: transfection with Src-shRNA affected the proliferation of osteosarcoma cells measured by CCK-8: (a) the proliferation of the MG-63 cell line was not significantly inhibited; (b) the proliferation of the U2-OS cell line was inhibited by the transfection with Src-shRNA (NC, negative control). Supplementary Figure S5: transfection with Src-shRNA affected cell proliferation of osteosarcoma cell lines measured by immunofluorescence against BrdU. (a) and (c) the proliferation of the MG-63 cell line was not significantly inhibited; (b) and (d) the proliferation of the U2-OS cell line was inhibited (NC, negative control; ^*∗*^*P* < 0.05; ×200). Supplementary Figure S6: cell cycle distribution measured by flow cytometry. Src-shRNA transfection induced a cell cycle arrest in the G1/S phase of the osteosarcoma U2-OS cell line but did not affect the cell cycle in the MG-63 cell line (^*∗*^*P* < 0.05; NC, negative control). Supplementary Figure S7: transfection with Src-shRNA in MG-63 and U2-OS cells inhibited cell invasion measure by Transwell assay. (a) The cells were observed and photographed again 24 hours later in different groups with a microscopic view (×400); (b) the cell counting in different groups (^*∗*^*P* < 0.05; NC, negative control). Supplementary Figure S8: transfection with Src-shRNA significantly inhibited migration of the osteosarcoma MG-63 and U2-OS cell lines. Osteosarcoma cells in the blank and negative control groups migrated to the midline significantly after 24 h, while the cell migration ability of Src-shRNA transfected cells was significantly reduced in MG-63 (a) and U2-OS (b) cell lines (NC, negative control; ×200). Supplementary Figure S9: transfection with Src-shRNA affected apoptosis of osteosarcoma cells. Src-shRNA transfection induced apoptosis in the U2-OS cell line but not in the MG-63 cell line. (a) Flow cytometry; (b) cell counting (NC, negative control). Supplementary Figure S10: tumor formation in nude mice. (a) Inoculated in the tibia bone marrow cavity of the left hind leg with osteosarcoma cells by the suspension method; (b) at the fourth week after inoculation, vascular engorgement of the skin surface of the tumor was observed. Supplementary Figure S11: confirmed osteosarcoma growth *in situ*. (a) The section of tumor showed a white fish shape; (b) the tumor invaded the tibia and part of the fibula was eroded and missing; (c) tumor grow *in situ* confirmed by X-ray film; (d) osteosarcoma tissue confirmed by HE staining (×200).
