# Supplementary material for: Tanshinone IIA Inhibits Osteosarcoma Growth through a Src Kinase-Dependent Mechanism
Source: Evid Based Complement Alternat Med. 2021 Jun 30;2021:5563691. doi: 10.1155/2021/5563691 (PMC8376467; doi:10.1155/2021/5563691)

### Supplementary materials

**Supplementary figure 1: Figure S1**


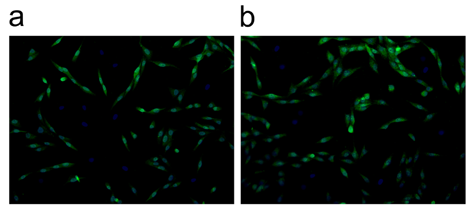


**Supplementary figure 2: Figure S2**


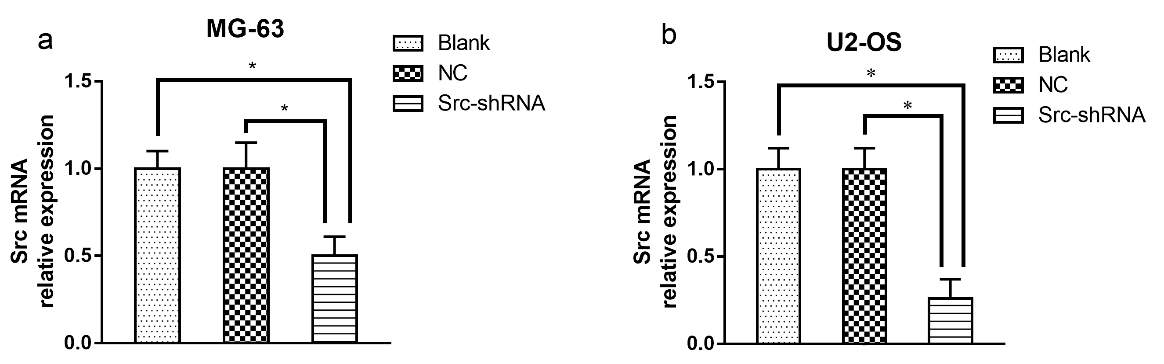


**Supplementary figure 3: Figure S3**


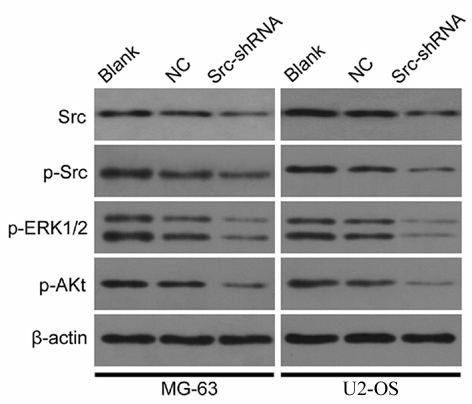


**Supplementary figure 4: Figure S4**


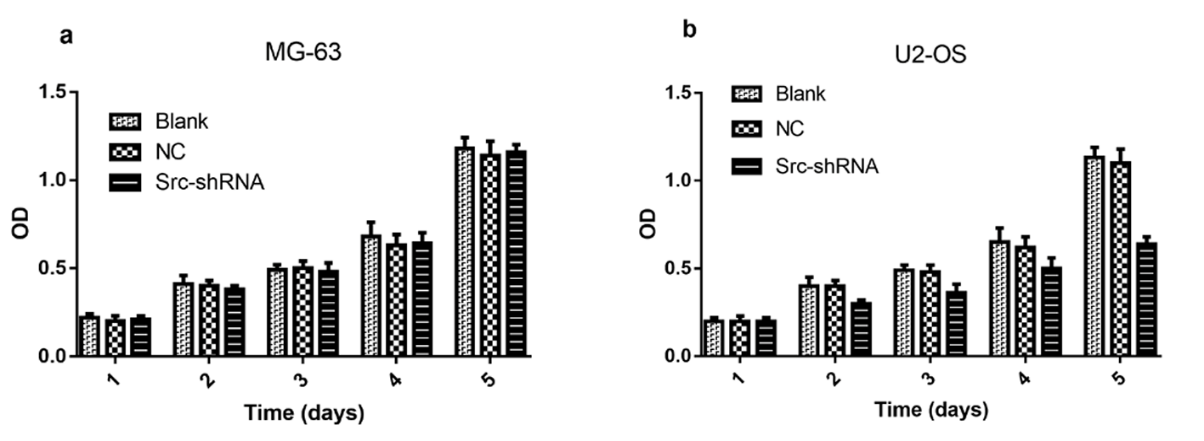


**Supplementary figure 5: Figure S5**


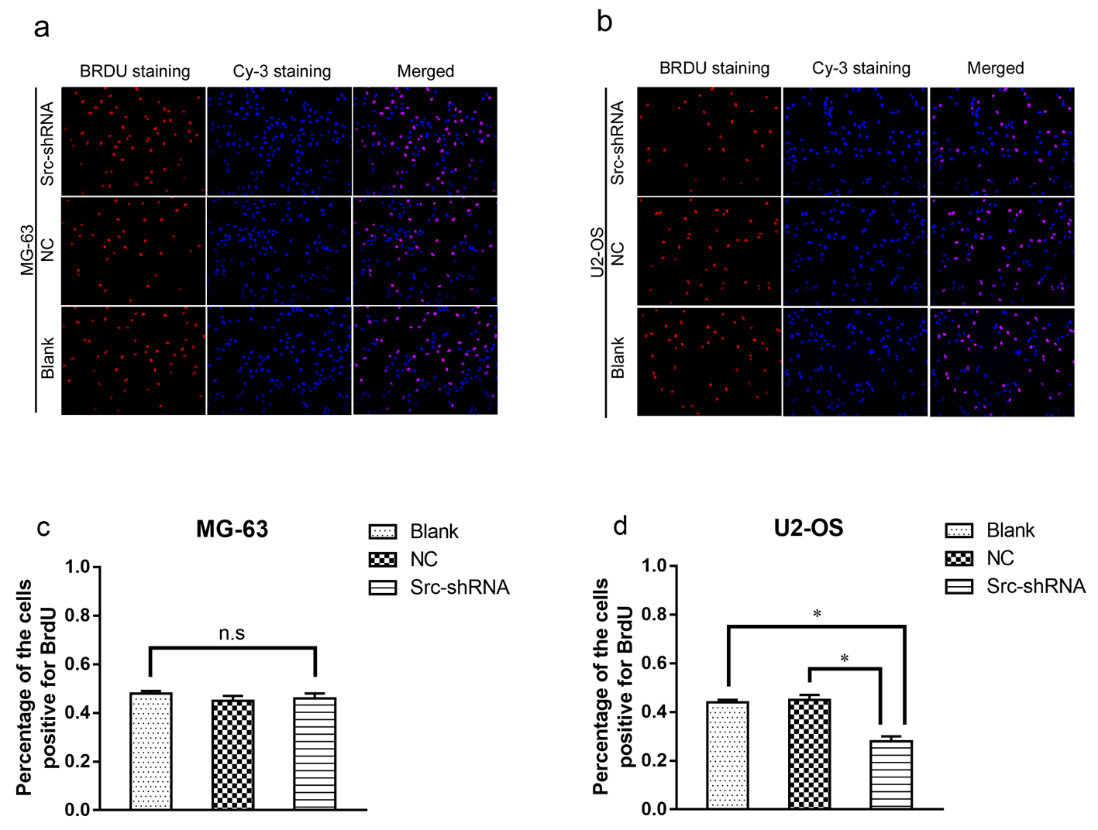


**Supplementary figure 6: Figure S6**


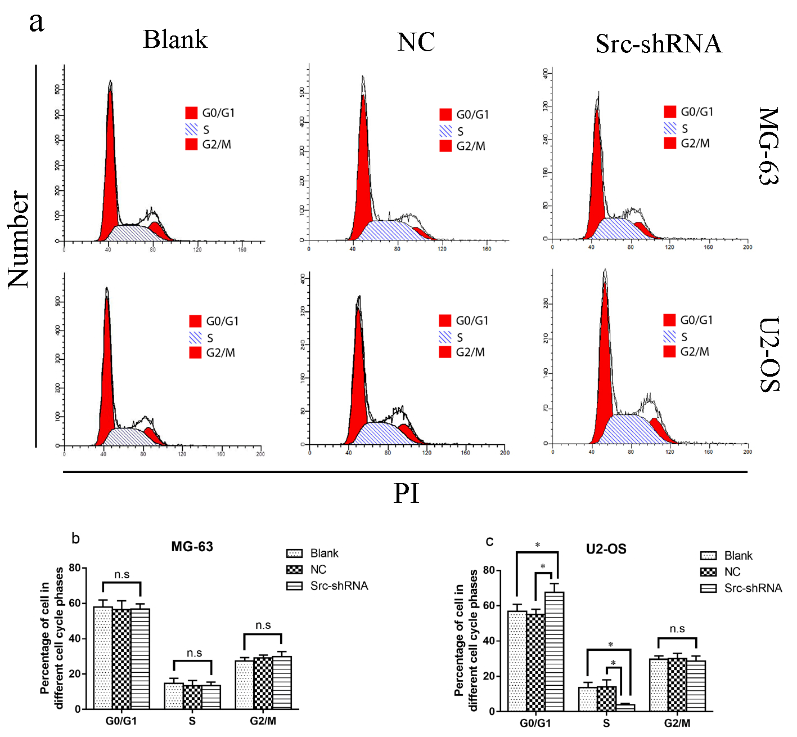


**Supplementary figure 7: Figure S7**


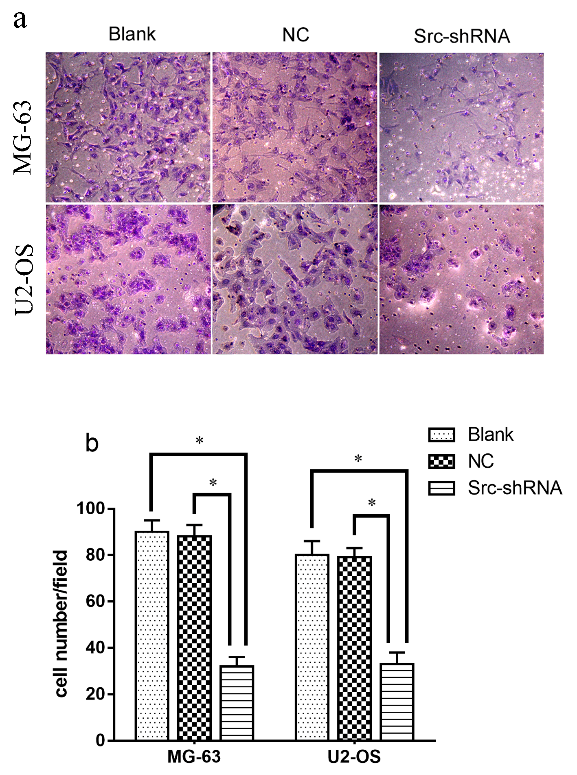


**Supplementary figure 8: Figure S8**


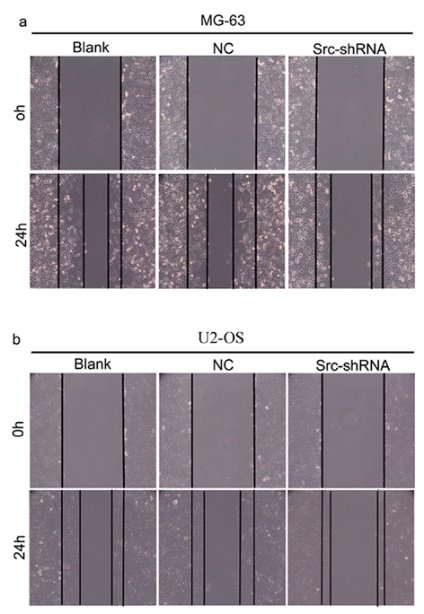


**Supplementary figure 9: Figure S9**


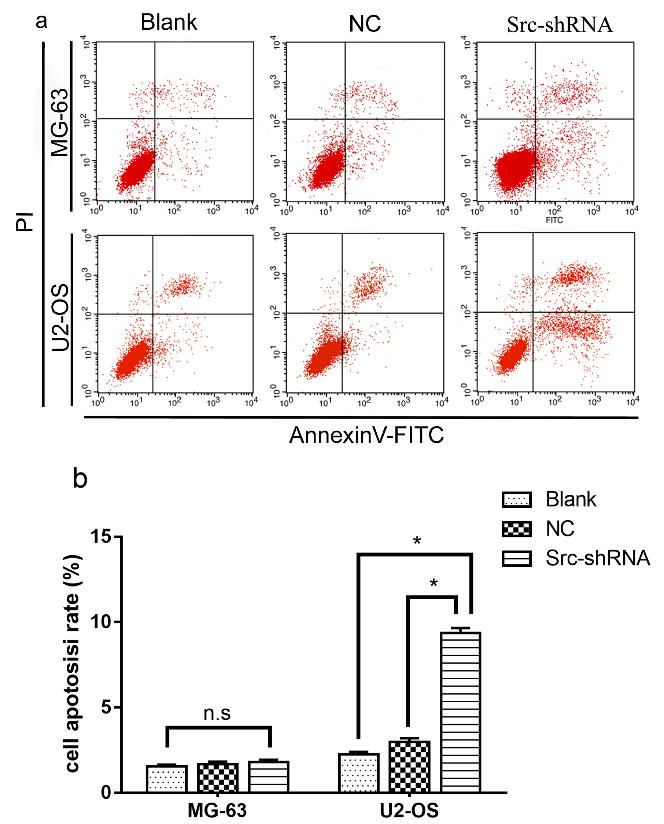


**Supplementary figure 10: Figure S10**


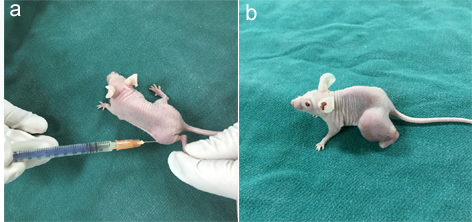


**Supplementary figure 11: Figure S11**


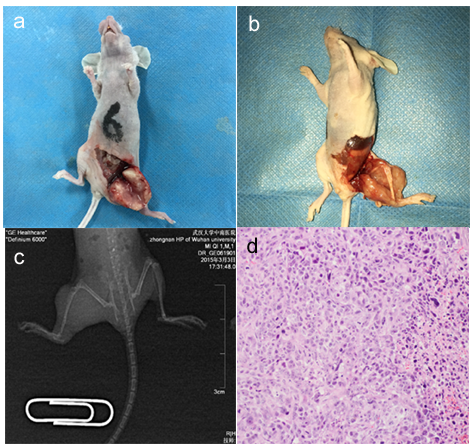

Supplement: Supplementary Materials. — Supplementary Figure S1: GFP-labeling of stable Src-shRNA-transfected osteosarcoma cells. The cells were shown in green, and the nuclei labeled by DAPI were shown in blue. (a) MG-63 cell line; (b) U2-OS cell line (×200). Supplementary Figure S2: transfection with Src-shRNA into osteosarcoma cells affected the expression of Src mRNA measured by RT-PCR. The expression of Src mRNA decreased in the MG-63 and U2-OS cell line (NC, negative control. ∗P < 0.05). Supplementary Figure S3: the effect of transfection with on the expression of Src, p-Src, p-ERK1/2, and p-AKt in osteosarcoma cells measured by Western blot. The expressions of the proteins were inhibited (NC, negative control). Supplementary Figure S4: transfection with Src-shRNA affected the proliferation of osteosarcoma cells measured by CCK-8: (a) the proliferation of the MG-63 cell line was not significantly inhibited; (b) the proliferation of the U2-OS cell line was inhibited by the transfection with Src-shRNA (NC, negative control). Supplementary Figure S5: transfection with Src-shRNA affected cell proliferation of osteosarcoma cell lines measured by immunofluorescence against BrdU. (a) and (c) the proliferation of the MG-63 cell line was not significantly inhibited; (b) and (d) the proliferation of the U2-OS cell line was inhibited (NC, negative control; ∗P < 0.05; ×200). Supplementary Figure S6: cell cycle distribution measured by flow cytometry. Src-shRNA transfection induced a cell cycle arrest in the G1/S phase of the osteosarcoma U2-OS cell line but did not affect the cell cycle in the MG-63 cell line (∗P < 0.05; NC, negative control). Supplementary Figure S7: transfection with Src-shRNA in MG-63 and U2-OS cells inhibited cell invasion measure by Transwell assay. (a) The cells were observed and photographed again 24 hours later in different groups with a microscopic view (×400); (b) the cell counting in different groups (∗P < 0.05; NC, negative control). Supplementary Figure S8: transfection with Src-sh [file 5563691.f1.docx]
